# Chronic In Vivo Effects of Repeated Exposure to Low Oral Doses of Tetrodotoxin: Preliminary Evidence of Nephrotoxicity and Cardiotoxicity

**DOI:** 10.3390/toxins11020096

**Published:** 2019-02-06

**Authors:** Andrea Boente-Juncal, Carmen Vale, Manuel Cifuentes, Paz Otero, Mercedes Camiña, Mercedes Rodriguez-Vieytes, Luis Miguel Botana

**Affiliations:** 1 Departamento de Farmacología, Farmacia y Tecnología Farmacéutica, Facultad de Veterinaria, Universidad de Santiago de Compostela, Lugo 27002, Spain; andrea.boente.juncal@usc.es (A.B.-J.); paz.otero@usc.es (P.O.); 2 Departamento de Anatomía, Facultad de Veterinaria, Universidad de Santiago de Compostela, Lugo 27002, Spain; m.cifuentes@usc.es (M.C.); 3 Departamento de Fisiología, Facultad de Veterinaria, Universidad de Santiago de Compostela, Lugo 27002, Spain; merchi.camina@usc.es (M.C.); mmercedes.rodriguez@usc.es (M.R.-V.)

**Keywords:** tetrodotoxin, toxicity, European mollusks, chronic exposure, risk assessment

## Abstract

Tetrodotoxin (TTX) is one of the most potent naturally occurring neurotoxins. Initially TTX was associated with human food intoxications in Japan, but nowadays, concerns about the human health risks posed by TTX have increased in Europe after the identification of the toxin in fish, marine gastropods, and bivalves captured in European waters. Even when TTX monitoring is not currently performed in Europe, an acute oral no observable effect level (NOAEL) of 75 μg/kg has been recently established but, to date, no studies evaluating the chronic oral toxicity of TTX have been released, even when EFSA has highlighted the need for them. Thus, in this work, the chronic effects of low oral TTX doses (below the acute lethal dose 50) were evaluated following internationally adopted guidelines. The results presented here demonstrate that low oral doses of TTX have deleterious effects on renal and cardiac tissues. Moreover, alterations in blood biochemistry parameters, urine production, and urinalysis data were already detected at the oral dose of 75 µg/kg after the 28 days exposure. Thus, the data presented here constitute an initial approach for the chronic evaluation of the in vivo toxicity of tetrodotoxin after its ingestion through contaminated fishery products.

## 1. Introduction

Tetrodotoxin (TTX) is one of the most potent naturally occurring neurotoxins. The toxin is produced by endosymbiotic bacteria including *Vibrio* sp., *Pseudomonas* sp., *Photobacterium* sp., *Aeromonas* sp., *Alteromonas* sp., *Bacillus* sp., *Micrococcus* sp., *Acinetobacter* sp., and *Moraxela* sp. [1], linked to the presence of *Prorocentrum minimum* [2], and also described in many species of pufferfish and gastropods [3,4,5,6]. Initially, TTX was associated with marine food intoxications mainly in Japan and Asia [7], but nowadays it has been reported in several European countries. At present, TTX is considered an emergent toxin in European waters [8,9]. Concerns about the health risks posed by TTX have raised in the last years, since TTX has been found in certain fish species like pufferfish, but also in marine gastropods and bivalves captured in European waters [3,9,10,11,12,13]. Moreover, several reports demonstrated that, mainly due to global warming and anthropogenic activity, TTX has currently expanded worldwide, and affects countries such as Greece, UK, Portugal, and Spain [10,11,13,14]. Besides bacteria and puffer fish, other species known to accumulate TTX include gastropods, newts, crabs, frogs, sea slugs, star fishes, blue-ringed octopuses, and ribbon worms [7,9,10,11,13]. Interest in this potent neurotoxin has also been raised by the success of clinical trials evaluating the analgesic effect of subcutaneous injections of TTX for the treatment of inadequately controlled moderate to severe cancer-related pain, although severe adverse effects including mainly nausea, dizziness, and oral hypoesthesia were reported after TTX treatment for 4 consecutive days [15].

The toxic action of TTX is due to its effect on voltage-dependent sodium channels (Na_v_) in excitable cells, selectively blocking six out of nine known Na_v_ isoforms at nanomolar concentrations, and therefore sodium channel entry into the cells and action potential generation [16]. Thus, avoiding action potential propagation and cell membrane depolarization, TTX immobilizes excitable tissues (nerves and muscle) and leads to neurological, cardiac, and gastrointestinal symptoms. Human symptoms caused by ingestion of TTX-contaminated food include tingling of the tongue and lips, headache, vomiting, muscle weakness, ataxia, and even death due to respiratory and/or heart failure. Currently there is no known antidote for TTX poisoning, and only supportive care is available [17].

Due to the worldwide expansion of TTX, the European Food Safety Authority (EFSA) recently published a scientific opinion on the risks related to the presence of TTX and analogues in marine bivalves and gastropods [18]. In this context, acute oral toxicity studies in mice established an oral lethal dose 50 (LD_50_) of 232 μg/kg TTX per body weight (BW), and a no observable effect level (NOAEL) of 75 μg/kg after monitoring the mice for two hours [19]. According to the current EU legislative requirements (Regulation 853/2004/EC and Regulation 854/2004/EC), fishery products derived from poisonous fish of the families Tetraodontidae, Molidae, Diodontidae, and Canthigasteridae (which are the main species containing TTX) must not be placed on the market [20], while in Japan, a regulatory limit of 2 mg eq TTX/kg was established [17], and recently TTX was formally included in a Dutch fish monitoring program which established a safe concentration of 44 µg TTX per kg of shellfish [21]. The geographical expansion of TTX and its detection in edible shellfish requires a risk assessment process leading to a safe seafood level for the toxin, in order to protect the health of consumers. However, to date, no chronic studies evaluating the oral toxicity of TTX have been released, even though EFSA has highlighted the need for chronic toxicity studies [18]. Therefore, the aim of the present work was to analyze the in vivo oral toxicity of TTX after repeated exposures, following the guidelines of the Organization for Economic Co-operation and Development (OECD) for the testing of chemicals [22], thus providing an initial approach to the chronic effects of this potent marine neurotoxin not yet currently monitored in Europe.

## 2. Results

### 2.1. Symptoms and Mortality Elicited in Mice by Repeated Oral Exposure to TTX

For toxicity evaluation, TTX was administrated by gavage daily for a period of 28 days at doses ranging from 25 to 125 µg/kg. The initial dose of 25 µg/kg of TTX was selected as the starting point because it was the lowest sub-lethal dose proven in acute oral studies to not cause adverse symptoms [19]. Dosing levels were subsequently increased to 75 µg/kg and 125 µg/kg. The same procedure was performed in control mice, which received the same volume of toxin solvent only.

Animals dosed with 25 µg/kg of TTX survived the entire experiment, and did not show any behavioral alteration or toxicity signs. Nevertheless, daily exposure of mice to 75 µg/kg of TTX caused the death of 2 out of 4 animals at days 1 and 5 after sudden cardiorespiratory failure few minutes after dosage, while no deaths were observed at this dose in the previous acute oral study with single toxin doses [19]. The next TTX dose evaluated was 125 µg/kg, and at this dose 2 out of 5 mice died at days 3 and 7 of treatment. Similarly to the effects of the 75 µg/kg TTX dose, the death of mice fed with 125 µg/kg TTX occurred rapidly after TTX administration (1 to 2 min), and only one animal suffered convulsions before sudden death. Survival times and mortality are summarized in Table 1.

Animals were observed daily during the study, and more closely during the first two hours after dosage. As mentioned above, no signs of toxicity occurred at the lowest dose of 25 µg/kg of TTX, while immediate symptoms occurring between 1 and 10 min after feeding, such as convulsions followed by cardiorespiratory failure, were observed immediately in two mice after 1 and 5 days of dosage with 75 µg/kg of TTX. At 125 µg/kg, two mice also suffered sudden death. At this dose, the first animal died on day 3 without behavioral signs of toxicity, showing only convulsions that started 1 min post administration. The second mouse, which died on the 7th day of treatment, presented a severe spinal curvature and piloerection starting on day 6, and on the day 7, immediately after TTX administration, started suffering convulsions and died shortly thereafter by cardiorespiratory failure. During the study, no gastrointestinal symptoms, paralysis, or incoordination were observed. All mice that died spontaneously were necropsied in order to exclude lung damage by gavage administration of TTX.

### 2.2. Effect of TTX on Mice Body Weight

Measurements of body weight and feed consumption are commonly used as an index of the toxicity of chemical compounds. As shown in Table 2, the effects of the 28 day repeated exposure of mice to each of the three TTX doses (25, 75, and 125 µg/kg) on BW and the corresponding accumulated weight gain were measured weekly during treatment at day 0 (first day of treatment), day 7, day 21, and day 28 (day of euthanasia, one day after the last TTX dose). No significant changes in BW were observed during the 28 days of treatment, however the absence of significant differences in BW could be due to the small number of animals employed in each group, since mice in the control group gained 3 g BW during the treatment, while mice in the group treated with 125 µg/kg TTX lost 1 g during the same period.

### 2.3. Effect of Repeated Oral Exposure to TTX on Faeces and Urine Production and Food Intake

The effects of the 28 day repeated exposure of mice to TTX at doses of 25, 75, and 125 µg/kg on feces and urine production as well as on feed consumption are summarized in Table 3. In order to evaluate these parameters, animals were placed individually, after the last dose at day 27, in metabolic cages for the last 24 h with free access to food and water until euthanasia at day 28. This procedure allowed monitoring of food consumption and sampling of feces and urine production for further biochemical analysis. As indicated in Table 3, no significant differences in feces production and food consumption were observed between TTX-treated mice and control mice. However, after the 28 day treatment, repeated administration of TTX significantly decreased daily urine production at the doses of 75 and 125 µg/kg. For instance, mice treated with 25 µg/kg of TTX had a reduction of urine production versus control group of only 2.6 mL, while mice treated with TTX at doses of 75 and 125 µg/kg showed a decrease in urine production of about 3.6 mL, which represents a 87.8% decrease, suggesting that repeated exposure of mice to TTX at doses as low as 75 and 125 µg/kg could affect renal function.

### 2.4. Effect of 28 Day Repeated Exposure of Mice to TTX on Blood and Urine Biochemical Parameters

**Figure 1:**
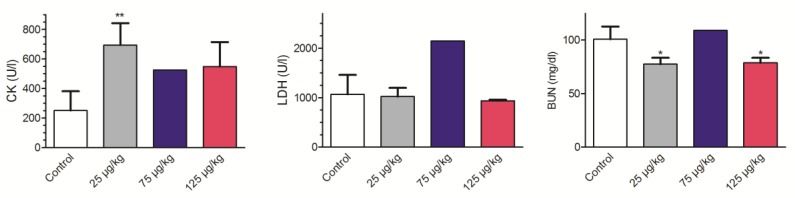
Effects of 28 days repeated exposure of mice to 25, 75 and 125 µg/kg of TTX on blood levels of creatine kinase (left), lactate dehydrogenase (middle) and BUN (right). Data are expressed as mean ± SEM of 3 to 5 determinations or mean (*n* = 2) for the dose of 75 µg/kg. * *p* < 0.05, ** *p* < 0.01. Creatine kinase (CK); lactate dehydrogenase (LDH); blood urea nitrogen (BUN).

Biochemical analysis of blood extracted by cardiac puncture was performed in samples from control and TTX-treated mice, in order to analyze the potential chronic effects of low doses of TTX. Blood parameters analyzed included electrolyte levels (Cl, Na and K), alanine transaminase (ALT), aspartate transaminase (AST), lactate dehydrogenase (LDH), creatine kinase (CK), blood urea nitrogen (BUN), glucose, and cholesterol. No effects of daily TTX administration on either blood glucose, cholesterol, ALT, or AST were observed at any of the toxin doses evaluated (Supplementary Figure S1). However, in spite of the small number of animals for which blood samples were available, alterations in blood biochemistry levels were observed for CK, LDH, and BUN (Figure 1). Thus, in mice dosed daily with 25 µg/kg TTX, CK increased from 250.4 ± 58.4 U/L (*n* = 5) in control animals to 694.0 ± 85.7 (U/L) in mice treated with 25 µg/kg TTX (t = 4.437; df = 6; *p* = 0.0044), to 525.0 in mice treated with 75 µg/kg TTX, and to 548.5 ± 117.5 (*n* = 3; t = 2.572; df = 5; *p* = 0.049) at the highest TTX dose. In spite of the CK increase in the blood of mice treated with TTX, it is noteworthy to indicate that, in all cases, its levels were in the physiological range (68 to 1070 U/L). Moreover, the blood levels of l LDH were significantly increased at the dose of 75 µg/kg and reached values of 1068 ± 159.3 U/L in control animals (*n* = 5) and 2143 (*n* = 2) in mice treated with 75 µg/kg of TTX, although in both cases LDH levels were again within the physiological range (1.105–3.993 U/L). Finally, BUN was decreased from 22.80 ± 1.1 mg/dL in control animals (*n* = 5) to 18.33 ± 0.8 mg/ dL in mice treated with TTX at 25 µg/kg (*n* = 3; t = 2.859; df = 6; *p* = 0.029), and to 18.67 ± 1.2 mg/ dL (*n* = 3; t = 2.47; df = 6; *p* = 0.048) in mice dosed 28 days with 125 µg/kg of TTX. No changes in BUN were observed in two blood samples obtained from mice dosed with the toxin at 75 µg/kg. Furthermore, no changes in blood electrolyte levels were observed at any of the TTX doses evaluated (Supplementary Figure S2). Altogether, these data suggest that repeated exposure to low doses of TTX may cause small alterations on blood physiological parameters in absence of overt toxicity signs.

As mentioned above, mice treated with TTX had a decreased urine production during the 24 h period after the last toxin dose, and therefore urine parameters were evaluated after free catch collection of urine during this interval. The low amount of urine collected at the highest toxin doses (Table 3) prevented the evaluation of urine parameters at shorter interval levels. Urine parameters analyzed in control and TTX-treated mice included color, clarity, specific gravity, protein, glucose, ketones, blood/hemoglobin, bilirubin, and urobilinogen. As indicated in Table 4, repeated exposure of mice to low doses of TTX, besides changes in urine production, led to changes in urine characteristics, with these effects being more evident in mice treated with the dose of 125 µg/kg TTX. Noteworthily, urine color changed from amber or yellow to darker colors in mice receiving the higher TTX doses. Urine turbidity also increased in mice treated with TTX at doses of 75 and 125 µg/kg, suggesting the possible presence of cells (leukocytes, erythrocytes, epithelial cells), crystalluria, or microorganisms. In addition, urine specific gravity increased in animals treated with higher toxin doses, in agreement with the increment of proteins and glucose levels. Furthermore, moderate ketonuria, bilirubinuria, and urobilinogenuria were also present in the animals treated with 125 µg/kg TTX, suggesting that renal function could be altered in these animals.

### 2.5. Anatomopathological Examination

After the 28 days of treatment, control and TTX-treated mice were euthanized, and detailed necropsy was realized, including macroscopic visualization of organs (heart, lungs, liver, kidneys, spleen, stomach, duodenum, rectum, and cerebrum). Macroscopically, animals did not present any evidence of organ damage, besides one mouse treated with TTX at 125 µg/kg that showed jaundice content in small intestine. This mouse died at day 7 of treatment after suffering fatal convulsions one minute post administration. Another mouse treated with the same toxin dose presented hemorrhage (blood clots) in the chest adhered in lungs on the day of euthanasia.

### 2.6. Histological Analysis of Organs from Control and TTX-Treated Mice

After euthanasia, samples of heart, liver, lungs, kidneys, spleen, stomach, duodenum, rectum, and cerebrum were immediately collected and fixed for staining with hematoxylin–eosin (H&E) or periodic acid–Schiff (PAS). Samples of all mice were analyzed in order to find histological alterations, however, only one representative image of the control and toxin-treated tissues is depicted.

**Figure 2:**
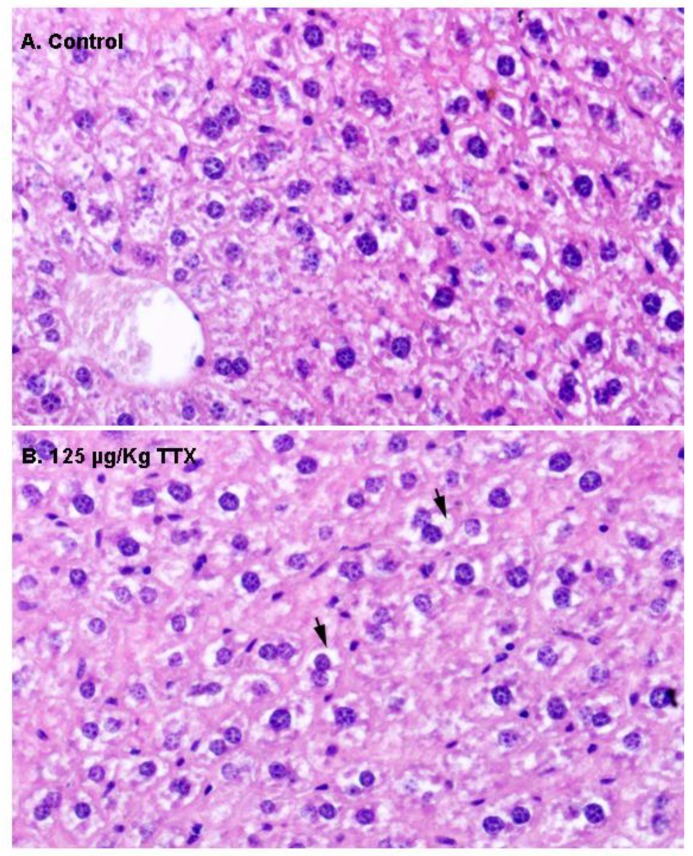
Representative hematoxylin–eosin (H&E) staining of the liver parenchyma in a sample from a control mouse (**A**), and from a mouse treated with 125 µg/kg oral TTX for 28 days (**B**). Compared to hepatocytes from control animals, more cytoplasmic vacuoles (non-stained spaces, indicated by arrows) were present in the hepatocytes from TTX-treated mice. In both cases, 600× microscope magnifications are shown.

First, liver samples were stained with H&E (Figure 2). In contrast to liver sections from control mice, in which hepatocytes showed an eosinophilic cytoplasm and a round nucleus (Figure 2A), hepatocytes in the livers of mice fed daily with TTX at 125 µg/kg during 28 days showed a more vacuolated (non-stained) cytoplasm (Figure 2B) that could represent cellular swelling, lipid accumulation, or glycogen stores.

**Figure 3:**
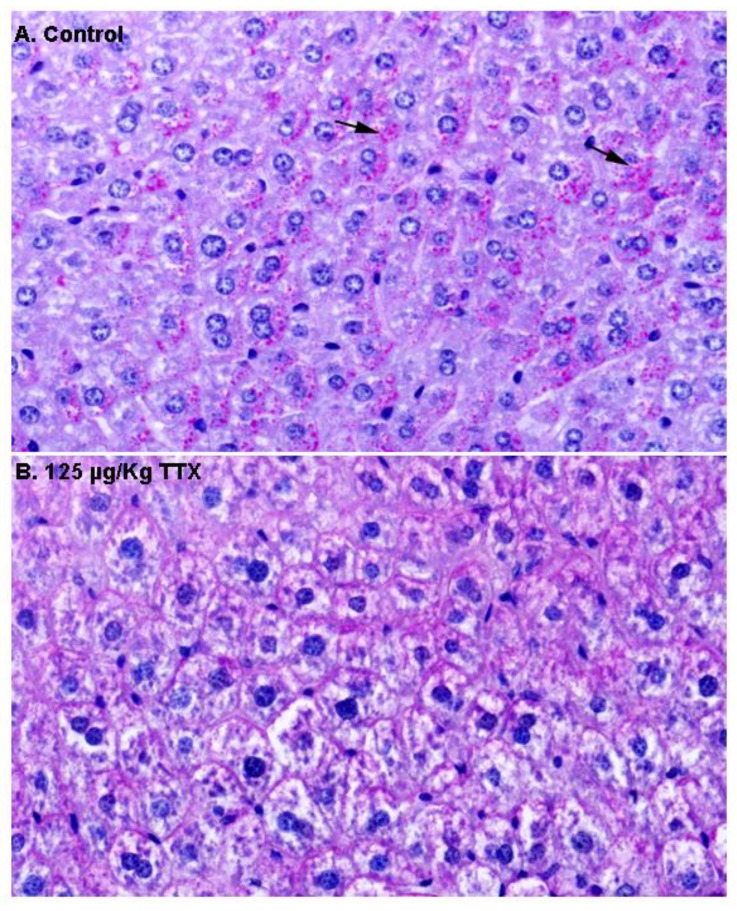
Periodic acid–Schiff (PAS) stained liver sections from control mice and mice treated with TTX at 125 µg/kg (600× magnification). Hepatocytes from control animals were PAS positive, with glycogen (magenta granules indicated by the arrows) located in the cytoplasm (**A**), while these granules were not present in the hepatocytes from mice dosed daily with 125 µg/kg TTX (**B**).

Next, in order to gain more insight into the content of these vacuoles, additional liver sections from control and TTX-treated mice were stained with PAS (Figure 3). As shown in Figure 3A, hepatocytes from control mice were strongly stained with PAS, and magenta granules (indicated by the arrows) considered to represent cytosolic glycogen were observed, while these granules were less evident in hepatocytes from TTX-treated mice (Figure 3B).

**Figure 4:**
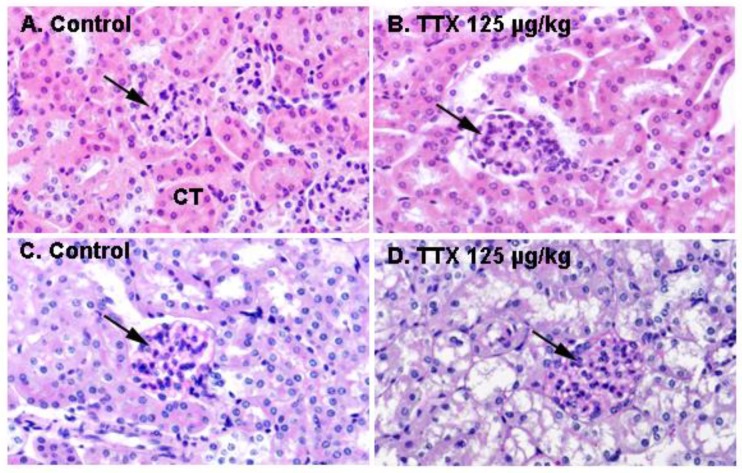
Kidney sections of a control mouse (left column) and of a mouse treated with 125 µg/kg TTX (right column), stained with H&E (**A**,**B**) or PAS (**C**,**D**). Renal corpuscules (arrows) and convoluted tubules (CT) are shown in the respective microscope magnifications at 600×.

In view of the effects of repeated oral administration of TTX on urine production and the above described alterations in urine parameters, kidney sections from control animals and from mice treated daily with TTX at 125 µg/kg were analyzed next. Figure 4 shows H&E staining of the kidney in control and TTX-treated mice. No evident histological alterations were observed by light microscopy in the kidney of TTX treated-mice.

**Figure 5:**
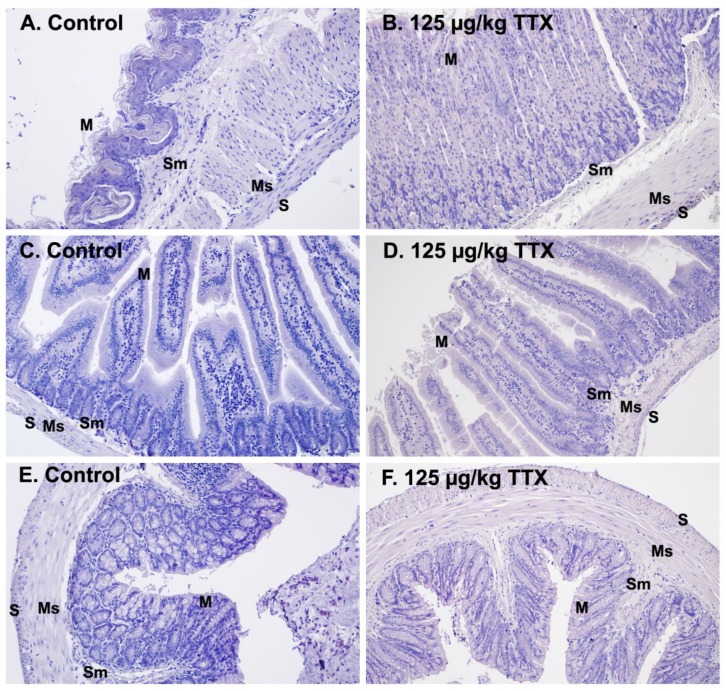
Representative light microscope micrographs obtained after H&E staining of the stomach (**A**,**B**), small intestine (**C**,**D**), and large intestine (**E**,**F**) from control mice (left panels) and mice treated daily with TTX at 125 µg/kg (right panels). M: mucosa layer, Sm: submucosa layer, Ms: muscular layer S: serosa layer. Microscope magnifications of 200× are shown.

As routine protocols recommend the analysis of several tissues [22], in order to obtain a good approach of the chemical toxicity, in the next series of experiments other main organs from control mice and from mice treated daily with 125 µg/kg of TTX were examined. For histological examination, the stomach was bisected longitudinally, and glandular and non-glandular regions were analyzed. As shown in Figure 5, H&E staining in control and TTX-treated animals showed unaltered mucosa with squamous keratinized epithelium, an intact keratin layer, and mucous cells. The submucosa layer containing vessels and the muscular layer oriented in different directions were also appreciated in both control and treated animals, and no apparent histological alterations were observed between control mice and TTX-treated mice (Figure 5A,B). Similarly, no changes were observed in the H&E staining of the small (Figure 5C,D) and large intestine (Figure 5E,F) of control and treated mice. Both intestines showed an unaltered basic structure formed by the mucosa and the submucosa layer, the smooth muscle layers, and the serosa layer. Identification of Brunner’s glands at higher magnifications confirmed that the sections shown in Figure 5C,D correlated to duodenum sections. Histological sections of large intestines with mucous cells and lymphoid tissue located in the submucosa in both control and TTX-treated animals are shown on Figure 5E,F.

**Figure 6:**
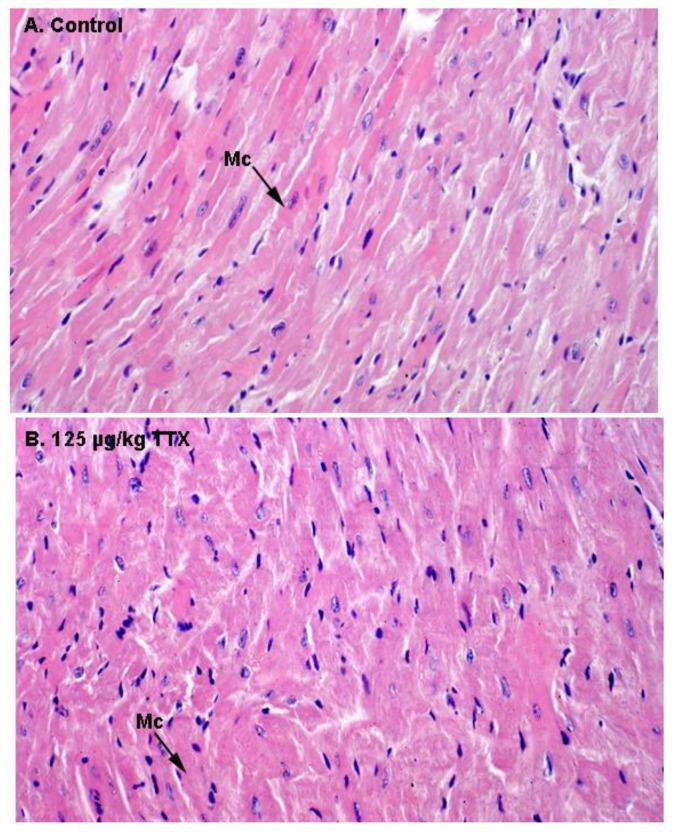
H&E stained sections of the myocardium by light microscopy. (**A**) Ventricle of control mouse. (**B**) Ventricle of a mouse treated with TTX at the dose of 125 µg/kg. Mc: Myocytes. Microscope magnifications of 400× are shown.

Finally, as indicate in Figure 6, histological alterations in the heart of control and TTX treated mice were evaluated by light microscopy. Representative images of H&E staining in heart sections of control and TTX-treated mice did not show any evidence of structural alterations in the heart. In both cases, normal cardiac myocytes, which are cylindrical in shape and have an elongated nucleus, were observed.

### 2.7. Ultrastructural Examination of Organ Damage Induced by Repeated Oral Dosing of Mice with TTX

**Figure 7:**
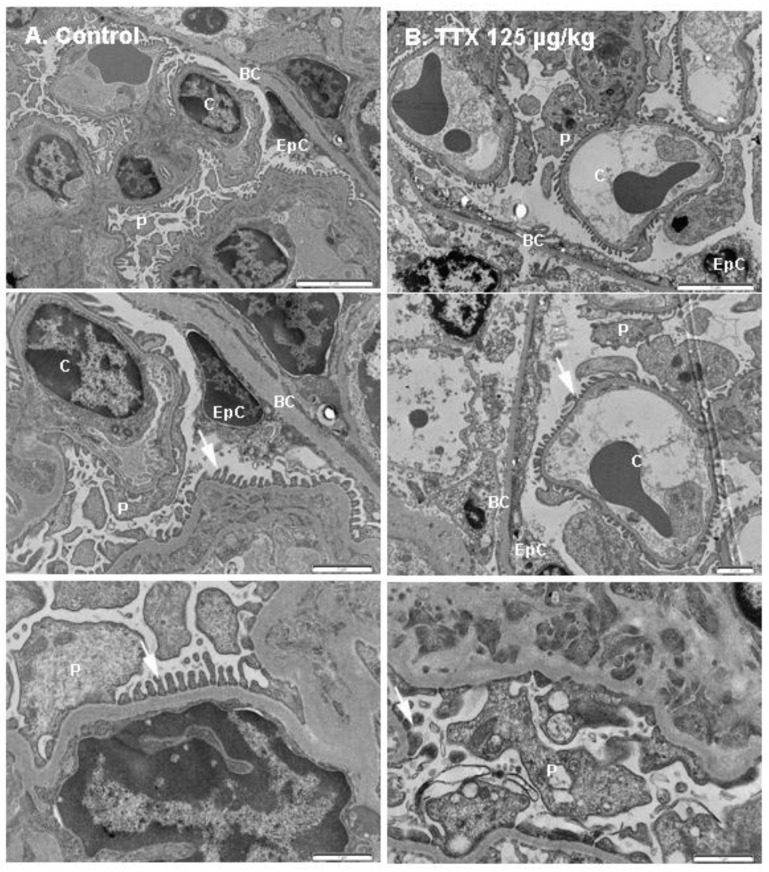
TEM micrographs showing kidney fields from (**A**) control mice (left panel) and from (**B**) mice treated with TTX at 125 µg/kg. Different magnifications are shown: scale bars are 5 µm (top panel), 2 µm (middle panel) and 1 µm (bottom panel). BC: Bowman’s capsule, C: capillary vessel, P: podocyte cell body, EpC: epithelial cell, arrows indicate the podocyte foot processes.

The following findings refer to the mice exposed daily to 125 µg/kg TTX that survived the whole treatment (28 days) and were sampled immediately after euthanasia. Control samples were obtained from mice that were fed by gavage with toxin solvent during the same period. First, and in view of the effects of TTX on urine production and urine parameters, transmission electron microscopic studies (TEM) of the kidney were performed. Since alterations in urine production pointed to changes in the renal filtration barrier or alterations in the reabsorption processes by epithelial cells of the proximal tubule, the kidneys of control and TTX-treated mice were evaluated to identify possible ultrastructural modifications on the glomeruli and the organization of the glomerular filtration barrier. These studies demonstrated evident ultrastructural changes in the kidney of TTX-treated mice (Figure 7). The renal filtration barrier, as shown in the schematic diagram depicted in Supplementary Figure S3, is composed of the capillary endothelial inner layer, the glomerular capillary basement membrane, and the podocyte layer. After TEM analysis, some evident changes in the glomeruli were detected in TTX-treated mice. In a normal basement membrane, between podocyte foot processes a consistent membrane gap called filtration slit membrane was observed (Figure 7A), which plays an important role preventing the passage of some molecules to the urinary space. This structure was altered in the kidney of animals treated with 125 µg/kg of TTX (Figure 7B). Under electron microscopy, other observed alterations included necrosis of the epithelial cells in the Bowman’s capsule, which showed irregular vacuolated cytoplasm and cell debris of necrotized podocytes. Moreover, endothelial capillary cells appeared damaged in the kidney of TTX-treated mice, with evident excess of cellular debris in the lumen of the capillary vessels.

**Figure 8:**
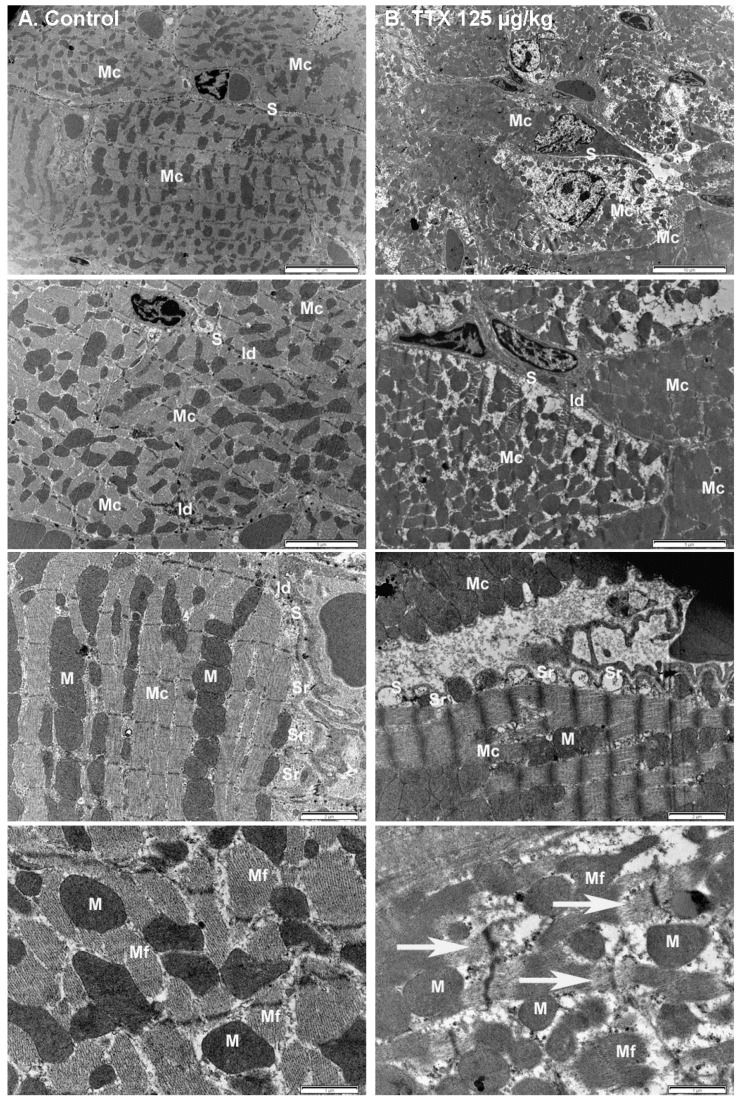
Electron micrograph of the myocardium from (**A**) control mice (left panel), and from (**B**) mice treated daily with 125 µg/kg TTX. Note the different magnifications shown. Scale bar is 10 µm, 5 µm, 2 µm, and 1 µm respectively, from top to bottom panels. Mc: myocardial cells, S: sarcolemma, Id: intercalated disk, Sr: sarcoplasmic reticulum, M: mitochondria, Mf: myofibrils. Evident multifocal vacuolization, sarcoplasmatic dilation, and disintegration of myofibrils (arrows in the bottom right panel) were observed in the heart of TTX-treated mice.

In as much as TTX-treated mice had higher levels of creatine kinase than control mice, the effect of TTX on the structure of cardiac muscle cells was also evaluated by TEM, focusing on the examination of myocardium. As shown on Figure 8, the typical cylindrical shape of myocardial cells and intercalated disks, as well as the prominent mitochondria depicted in the left panel, were normal in control animals. However, in mice treated daily with 125 µg/kg of TTX (right panel) ultrastructural analysis of the myocardium showed multifocal vacuolization around the sub-sarcolemmal area that extended inwards as the injury progressed. Another feature in treated mice was dilatation of the sarcoplasmatic reticulum, shown in the 2 µm micrograph. In longitudinal sections, degeneration of cardiac myofibrils was appreciated in high magnification images, as indicated by the arrows (lower right panel), showing structural disorganization and disintegration of myofibrils in TTX-treated mice.

### 2.8. Quantification of TTX in Blood, Faeces and Organs by LC-MS

**Figure 9:**
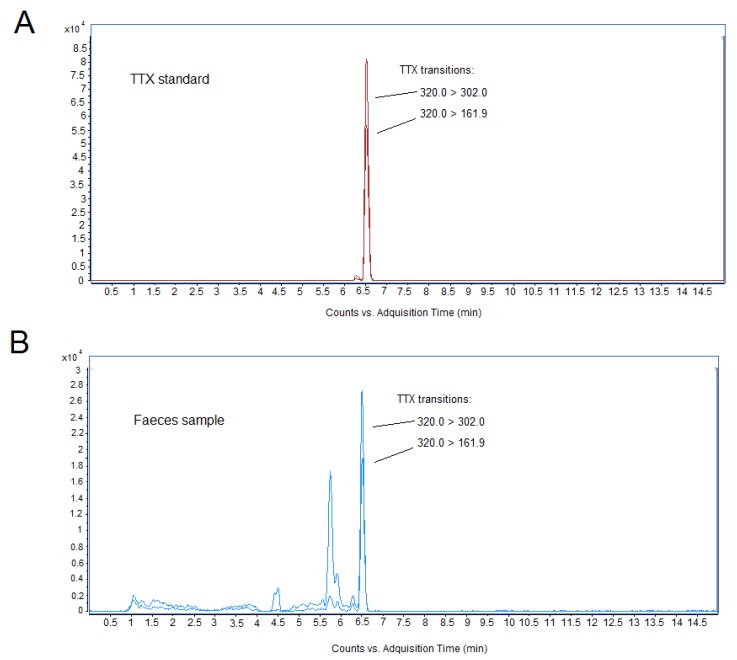
Liquid chromatography–mass spectrometry (LC-MS) chromatogram of TTX standard (**A**) and feces samples collected after 28 days repeated oral administration of 125 µg/kg of TTX to mice (**B**). Feces samples were collected for 24 h after the last toxin administration.

The identification and quantification of TTX in biological samples was performed by liquid chromatography–mass spectrometry (LC-MS) analysis. The quantity of TTX detected in biological samples is summarized in Table 5. After 28 day oral administration of 75 and 125 µg/kg of toxin, blood, feces, and representative organs such as kidney and liver samples were processed for toxin determination, since previous work has demonstrated that radiolabeled intramuscular TTX accumulated in liver and kidney [23,24]. All feces samples of mice dosed with 125 µg/kg TTX and recollected 24 h after the last toxin administration were positive for the toxin, indicating that the toxin was partially eliminated unaltered in feces (mean 145.9 ± 33.1 ng/g), and thus not absorbed. TTX concentrations in feces ranged between 61.31 and 254.67 ng/g. However, no TTX was detected in feces of animals fed with 75 µg/kg TTX, although the limit of detection (LOD) of the toxin was of very few micrograms. For a mouse fed with 125 µg/kg of TTX, the amount of toxin administered to a 20 g mice was 25 µg and, considering the amount of feces over a 24 h period, the amount of toxin excreted by feces represented about 6.38% of the TTX oral dose, thus suggesting that the toxin was likely widely absorbed in the intestinal tract and rapidly eliminated. Representative chromatograms for the LC-MS peak of the TTX standard and for TTX detected in feces samples from the 125 µg/kg treatment are shown in Figure 9. In addition, no toxin was detected in blood samples, nor in the organs of mice treated with 125 µg/kg of TTX, after the 28 day study, however, this could be due to the small size of the samples available for LC-MS analysis

## 3. Discussion

Currently, TTX has been reported in several European countries, and it is considered an emergent toxin in European waters [8,9], causing human intoxications due to the ingestion of contaminated fishery products. Recently, the oral LD_50_ for TTX was established as 232 µg/kg BW and the oral NOAEL as 75 µg/kg BW [19]; these values were reviewed by EFSA, leading to the establishment of a toxin level below 44 μg TTX equivalents/kg shellfish meat [18] as a safe concentration in fishery products. This limit of TTX in fishery products has already been officially adopted by the Netherlands [21], the first country in Europe that included this expanding toxin in their shellfish monitoring program. In contrast to the above LD_50_ for acute oral TTX, obtained from mice fed with a normal diet, a higher median oral LD_50_ of 1890 nmol/kg by gavage or 2850 nmol/kg by feeding mice with TTX in cream cheese has also been reported [25], however this high LD_50_ by feeding could result from the high fat content of the artificial diet. In fact, the same report also described larger death rates after oral administration of TTX than those previously reported after acute gavage administration of the toxin [19], and than the death rates reported in the present study. Care should be taken with the route of administration employed for oral determination of the LD_50_, since, in agreement with our previous work [19] and even when mice in the present report had free access to food throughout the chronic treatment, mortality and evident kidney and heart alterations were already observed at the 125 µg/kg dose (i.e., about 391 nmol/kg), in contrast with the data previously reported after TTX administration feeding mice with cream cheese [25].

During the last few years, an increasing number of reports have described the presence of TTX in shellfish and gastropods captured in European waters [1,5,9,10,13,14,21]. A recent study on the occurrence of TTX in the Netherlands reported TTX concentrations of 253 µg TTX/kg in oysters and 101 µg TTX/kg in mussels [21]. Moreover, TTX has been present in Greek shellfish since 2006, in concentrations ranging between 61.0 and 194.7 μg/kg [13], and in shellfish from the UK at concentrations as high as 137 µg TTX/kg, reaching 253 µg TTX/kg in oysters [11,12]. However, in a recent opinion of EFSA on TTX-related risks for humans, a major drawback regarding the lack of data on the subchronic or chronic toxicity of TTX and its analogues has been identified [18]. Therefore, the potential chronic effects of low oral TTX doses were evaluated in this study, following internationally accepted guidelines for the testing of chemicals [22]. In spite of the low number of animals that survived the 28 days of oral exposure to the higher TTX doses analyzed, which were chosen on the basis of previously reported oral acute doses that did not produce effects in mice [19], we described here unexpected chronic deleterious effects of TTX that may pose a risk for human health, especially after consuming seafood captured while fishing [26]. Even when the low number of animals precludes the use of this data to inform policy changes on TTX levels in fishery products, the study reports for the first time potentially harmful effects after repeated exposure to this neurotoxin.

Unexpectedly, daily oral administration of TTX doses of 75 µg/kg and 125 µg/kg to mice caused the death of some animals almost immediately after oral dosage, even when the mice were apparently healthy and without observed behavioral modifications, a fact that could be related to the previously reported higher concentrations of TTX in lungs than in plasma after intramuscular administration to rats [23]. Assuming that in mice TTX can also concentrate in lungs, this could cause the respiratory failure and rapid death after the oral administration of the toxin. Furthermore, the same report identified higher concentrations than the plasmatic concentration of toxin in the stomach, kidney, and intestines of rats. These observations are in agreement with the data presented here, since even though low TTX doses did not have a major effect on food consumption or body weight, the toxin almost completely suppressed urine production over a 24 h period in mice dosed with TTX at 75 µg/kg and 125 µg/kg. Moreover, the urinalysis was altered after repeated oral exposure of mice to TTX, with the effects being dose-dependent and manifested by darker urine colors and increasing turbidity, together with moderate ketonuria, bilirubinuria, and urobilinogenuria after 28 days oral administration of the toxin. Subtle alterations in blood biochemistry parameters were also identified, noticeably the increase in LDH and CK levels, however, even when these parameters were increased with respect to control animals, they were still within the normal physiological range.

Urinary excretion of TTX has been documented in humans and rats [23,27]. The results presented here indicate that repeated exposure of mice to TTX at doses of 75 and 125 µg/kg severely decreased urine production, which could be indicative of kidney injury. However, this effect could also be attributed to the recently reported effect of TTX decreasing the number of cholinergic nerve fibers in the urinary bladder wall, blocking muscle activity and leading to an antidiuretic effect when administered intravesically [28]. Nevertheless, the alterations in the urinalysis, most evident in animals treated with higher doses of TTX, suggested possible renal damage. As suspected, renal damage was confirmed after TEM observation of kidney samples, indicating that proteinuria was likely associated with an increased permeability of the glomerular filtration barrier. Electron microscope analysis of the kidney from mice dosed with 125 µg/kg of toxin showed extensive damage in the Bowman’s capsule, with necrotic podocytes and epithelial cells as well as altered podocyte foot processes. The role of voltage gated sodium channels in kidney physiology is evidenced by the fact that mutations in the Na_v_1.8 sodium channels are associated with human kidney stone disease [29], a fact that is in agreement with the observation that the predominant innervation in the kidney is attributed to neurons mainly containing the TTX-sensitive Na_v_1.7 and the TTX-resistant Na_v_1.8 and Na_v_1.9 isoforms of voltage-gated sodium channels [30]. In this work, we demonstrated that chronic exposure to TTX caused podocyte injury. Podocytes are responsible for maintaining the glomerular filtration barrier, and there is a demonstrated causal relationship between abnormalities of single podocyte molecules, proteinuria, and glomerulosclerosis [31]. Podocytes adapt to maintain homeostasis, but excessive stress leads to maladaptation, with complex biological changes including loss of integrity and dysregulation of cellular metabolism that ultimately cause proteinuria and detachment from the glomerular basement membrane. Podocytes are highly dependent on their actin cytoskeleton to stabilize their complex architecture. FERM-domain protein EPB4.1L5 links the actin cytoskeleton to cell membrane proteins, and its depletion from podocytes triggers proteinuria, foot process effacement, and early lethality from kidney disease [32]; therefore, this anchoring system could potentially be affected by TTX. Another target of TTX in glomerular disease could also be the mTOR pathway, which has been demonstrated to have an important role in podocyte homeostasis [33]. However, most likely, and since TTX has been reported to cause hypotension [34,35], this effect could also underlie the kidney lesions elicited by the long term exposure of mice to low oral doses of TTX. Nevertheless, a renal origin for the kidney lesions should also be considered, since proteinuria is the consequence of the increased permeability of the glomerular filtration barrier or the damage of the reabsorption mechanisms of the cells in the proximal tubule [36], allowing large molecules to pass into the urinary space and be excreted in urine. A previous in vivo study demonstrated that infusion of TTX in brain tissue produced a reduction in the activity and response of Na^+^,K^+^-ATPase [37], which is the most important channel in the basolateral membrane of proximal tubule cells and is highly expressed in collecting duct of rodents. Initial studies also indicated that intrarenal TTX in very low concentrations caused increased excretion of a number of inorganic ions, including sodium. In this case, one of the hypothesis raised by the authors was that if TTX blocked hypothetical ‘sodium channels’ normally open in the luminal membrane, decreased entry of sodium into proximal epithelial cells at their luminal borders would result, and decreased sodium reabsorption and thus natriuresis would be observed [38], however, this hypothesis needs still to be demonstrated. Moreover, although cardiac alterations have been reported in human intoxications with TTX [17,39], we describe here for the first time cardiac degeneration in mice treated daily with the toxin at 125 µg/kg. However, this effect is difficult to explain, since the tetrodotoxin-resistant Na_v_1.5 channel (blocked by micromolar concentrations of the toxin) is suggested to be the only functional voltage-gated Na^+^ channel in the adult, non-diseased myocardium of higher mammals [39,40]. Therefore, future experiments should be performed to evaluate the blood levels of TTX and its potential ability to accumulate, and thus affect Na_v_1.5 function leading to myocardial degeneration.

The results presented here constitute the first analysis of the potentially chronic effects of low oral doses of TTX. In summary, our study demonstrates dose-dependent mortality after chronic administration of TTX by gavage, and revealed cardiac and renal damage even at doses of 125 µg/kg BW. Renal and cardiac effects are also expected at the dose corresponding to the acute NOAEL after oral TTX administration, which has been established at 75 g/kg in view of the preliminary data reported here. However, further studies must be pursued with larger numbers of animals, in order to exactly determine the potential harmful effects of low oral doses of TTX.

## 4. Conclusions

Currently, TTX represents a main concern for food fishery security, since it has been reported in several European countries in recent years. Recently, the acute oral NOAEL for TTX has been established as 75 µg/kg BW. These values were reviewed by EFSA and led to the establishment of a safe concentration of TTX as below 44 μg TTX equivalents/kg shellfish meat in fishery products. However, the results obtained here indicate potential harmful effects of low oral doses of TTX that require further and detailed studies in order to establish the safety levels of this potent neurotoxin in fishery products.

## 5. Materials and Methods

### 5.1. In Vivo Experimental Procedure

In vivo studies were performed with Swiss female mice weighing 18–30 g (4 weeks old). All animal procedures were carried out in conformity with European legislation (EU directive 2010/63/EU) and Spanish legislation (Real Decreto 53/2013, Decreto 296/2008), and with the principles approved by the Institutional Animal Care Committee of the Universidad de Santiago de Compostela under procedure number 011/14, authorized on 14ugust 2014 (MR110250). The toxin used in this work was a solution of tetrodotoxin (CAS Number 4368-28-9) and 4,9-anhydro tetrodotoxin (CAS number 13072-89-4) in aqueous 1 mM AcOH solution (pH 3.91), which contained 25.9 ± 1.3 µg/g of tetrodotoxin and 2.99 ± 0.16 µg/g of 4,9-anhydro tetrodotoxin, together with a low level of 11-deoxy tetrodotoxin provided as certified reference material (code CRM-03-TTXs) by CIFGA laboratories (Lugo, Spain). Prior to administration, TTX was diluted in 0.9% saline solution to achieve each dose. Doses ranging from 25 to 125 µg/kg BW of TTX were administrated by gavage every 24 h over 28 days, reaching a final volume of 200 µL of solution per mouse. Control mice were fed with 200 µL of saline solution containing the corresponding amount of AcOH. Mice were observed intermittently during study and more closely during the first two hours after dosage. Animals had free access to food and water, they were housed in separate cages, one cage per toxin dose, and the toxin doses were administrated sequentially, starting at the lower dose and initiating the following dosage period after finishing the 28 day treatment. During treatment, mice were kept in rooms with constant temperature, humidity, and a controlled photoperiod at the animal facilities of the School of Veterinary Medicine of the University of Santiago de Compostela (Code: AE-LU-002). During experiments, animals were weighed weekly at day 0 (day of the fist treatment), day 7, day 14, day 21, and day 28, and food consumption was also registered at the same intervals. Euthanasia was performed on day 28. Moribund animals or animals obviously in pain or showing signs of severe and enduring distress were humanely killed, and considered in the interpretation of the test results in the same way as animals that died on the test. After the last TTX dosage (day 27), control and TTX-treated animals were placed in metabolic cages for 24 h in order to monitor urine and feces production. Finally, animals were euthanized in a CO_2_ chamber and blood and tissue samples were collected. All animals in the study were subjected to a full necropsy, including detailed visualization of heart, liver, lungs, kidneys, spleen, stomach, duodenum, rectum, and cerebrum.

### 5.2. Blood and Urine Analysis

Blood was extracted by cardiac puncture in the ventricle of euthanized mouse, using non heparinized needles and syringes. Immediately after extraction, blood samples were transferred to lithium heparin microtubes (2.5 U.I. LH/mL). After careful mixing for 30 s, blood was centrifuged for 90 s in a high speed microcentrifuge (Idexx Stat Spin 15800 rpm/12,000× *g*, IDEXX Europe B.V., Hoofddorp, The Netherlands). Samples with altered visual properties that could alter biochemical parameters (high hemolysis, icterus, or lipidemia) were not further processed. For blood analysis, the computer controlled biochemical analyzer Idexx Catalyst Dx (IDEXX VetLab Station, IDEXX Europe B.V., Hoofddorp, The Netherlands) was used. Briefly, 300 μL of plasma were used to evaluate electrolyte levels (Cl, Na and K), ALT, AST, LDH, CK, and BUN. Glucose and cholesterol were also determined. All the biochemical parameters were first analyzed in undiluted samples, and only in some cases in which the parameter was out of the reference range of the analyzer, an automatic dilution with physiological saline was performed. For urine analysis, a reflectance photometer (IDEXX VetLab UA, IDEXX Europe B.V., Hoofddorp, The Netherlands) which reads and evaluates IDEXX UA Strips was used to determine color, clarity, protein, glucose, ketones, blood/ hemoglobin, bilirubin, and urobilinogen. Urine specific gravity was measured with a refractometer.

### 5.3. Histological and Ultraestructural Analysis

After euthanasia, macroscopic examination was followed by preparation of the tissues for light microscopy and transmission electron microscopy (TEM). Samples of following organs: heart, liver, lungs, kidneys, spleen, stomach, duodenum, rectum, and encephalon were immediately collected and fixed by immersion in buffered Bouin’s solution (75% picric acid, 20% formaldehyde, 5% acetic acid) for 24 h at 4 °C, and then transferred to 70% alcohol. After fixation, samples were embedded in paraffin wax according to standard laboratory procedures, cut into sections of 3 µm thickness, and mounted into slides. Sections were stained with hematoxylin–eosin (H&E) or periodic acid–Schiff (PAS) for structural assessment of the tissues and examined under a light microscope. Digitalized images were obtained with an Olympus microscope digital camera DP74 coupled to an Olympus AX70 microscope.

Sample preparation for transmission electron microscopy (TEM) was performed according to previously described procedures [41]. In brief, organ samples (1 mm^3^) were fixed by immersion (2.5% glutaraldehyde in 0.1 M cacodylate trihydrate buffer) for 2 h at 4 °C and rinsed three times with 0.1 M cacodylate trihydrate buffer. Postfixation by immersion in 1% OsO4 in 0.1 M cacodylate trihydrate buffer was performed for 60 min. After, tissues were rinsed and dehydrated in graded ethanol solutions, including one bath with 70% ethanol and 0.5% uranyl acetate, rinsed in propylene oxide, and embedded in Epon 812 (Momentive Specialty Chemicals Inc., Houston, TX, USA). A Leica Ultracut UCT ultramicrotome (Leica Microsystems GmbH, Wetzlar, Germany) was used to obtain ultrathin sections of tissue samples. Samples were counterstained with uranyl acetate and lead citrate, and ultrastructural analysis of 1 mm^2^ thick samples was performed with a JEOL JEM-1011 Transmission Electron Microscope (Jeol Ltd., Tokyo, Japan).

### 5.4. Sample Preparation for LC-MS Analysis

TTX extraction from blood was performed according to previously described procedures [19,42]. A slightly modified protocol was applied for TTX extraction from feces, urine, and organs. Briefly, mouse blood (100 µL) or plasma (15–53 µL) was mixed with 800 µL of 2% acetic acid and vortexed for 5 min. The mixture was transferred to an ultrafiltration spin column (Amicon^®^ Ultra 3K centrifugal filters, Merck Millipore, Darmstadt, Germany) and centrifuged at 4000 rpm for 40 min. Mouse feces (0.3 g) were mixed with 1600 µL of 2% acetic acid and vortexed for 5 min, and the extract was centrifuged at 4000 rpm for 15 min. The supernatant was transferred to the ultrafiltration spin column and additionally centrifuged at 4000 rpm for 60 min. Mouse kidney (0.067–0.257g) and mouse liver (0.177–0.297g) were cut into small pieces (<1 mm^3^), mixed with 800 µL of 2% acetic acid and vortexed for 5 min, then extracted again with 800 µL of the same solution. Supernatants were pooled, filtered through 0.22 µm, transferred to an ultrafiltration spin column, and additionally centrifuged at 4000 rpm for 30 min. In all cases, ultrafiltered solutions were dried, dissolved in 200 µL of 0.03M acetic acid, and filtered again through 0.45 µm filters before placing then in the LC-MS vial for analysis. The same protocol was applied to blood, feces, and tissue samples from control animals.

### 5.5. LC-MS Analysis of TTX

Chromatography separation was carried out using a 1290 Infinity ultra-high-performance liquid chromatography system coupled to an Agilent G6460C Triple Quadrupole mass spectrometer equipped with an Agilent Jet Stream ESI source (Agilent Tecnologies, Waldbronn, Germany). TTX identification and quantification was performed according to Rodriguez et al. [43]. Briefly, toxin was separated using an ACQUITY UPLC BEH Amide column (2.1 × 100 mm, 1.7 µm, Waters, Manchester, UK) at 35 °C, with an injection volume of 5 µL. The composition of the mobile phase A was 10 mM ammonium formate and 0.1% formic acid in water, and mobile phase B was acetonitrile containing 0.1% formic acid and 2% 100 mM ammonium formate dissolved in water. Chromatographic separation was performed by gradient elution: starting with 95% B and reducing to 5% B over 11 min, then 5% B was held for 1 min, and increasing to 95% B over 1 min. Finally, 95% was held for 2 min until the next run (run time 15 min). The mass spectrometer was operated in positive mode, and analyses were performed in Multiple reaction monitoring (MRM) mode monitoring two transitions: *m*/*z* 320.0 > 161.9 (identification) and *m*/*z* 320.0 > 302.0 (quantification).

### 5.6. Statistical Analysis

Statistical analyses were carried out using GraphPad Prism 5, Version 5.01, ©1992-2007 GraphPad Software, Inc, La Jolla, CA, USA. All values are expressed as mean ± SEM. A *p*-value of <0.05 was considered statistically significant. ANOVA was used for multiple comparisons (weight changes, food consumption, feces, and urine) using repeated measures, followed by Dunnett comparisons test.

## Figures and Tables

**Table 1:** Mortality induced by the repeated dose 28 day oral toxicity study in mice after gavage administration of tetrodotoxin (TTX), and survival times corresponding to each treatment.

Dose (µg/kg)	Total Mice	Dead	Survival Time (Days)	Mortality %
Control	5	0	28	0
25	3	0	28	0
75	4	2	1, 5, 28, 28	50
125	5	2	3, 7, 28, 28, 28	40

**Table 2:** Effect of repeated exposure of mice to TTX on body weight (BW)and accumulated weight change during the 28 day treatment period. Values are expressed as mean ± SEM, except for the dose of 75 µg/kg, where results represent the mean (*n* = 2).

Group	Day 0	Day 7	Day 14	Day 21	Day 28
**Control**	(*n* = 5)	(*n* = 5)	(*n* = 5)	(*n* = 5)	(*n* = 5)
Body weight (g)	20.2 ± 0.5	20.6 ± 0.5	23.2 ± 1	23.5 ± 1.5	23.3 ± 2.1
Cumulative BW (g)	0.0	0.4 ± 0.6	3.0 ± 1.0	3.4 ± 1.5	3.2 ± 2.1
**25 µg/kg TTX**	(*n* = 3)	(*n* = 3)	(*n* = 3)	(*n* = 3)	(*n* = 3)
Body weight (g)	19.8 ± 0.5	20.7 ± 1.2	21.9 ± 1.1	22.2 ± 0.9	22.7 ± 0.5
Cumulative BW (g)	0.0	0.9 ± 0.9	2.1 ± 0.7	2.4 ± 0.8	2.9 ± 0.3
**75 µg/kg TTX**	(*n* = 4)	(*n* = 2)	(*n* = 2)	(*n* = 2)	(*n* = 2)
Body weight (g)	19.0 ± 0.6	19.8	21	22.1	23.0
Cumulative BW (g)	0.0	0.7	1.9	3.1	3.9
**125 µg/kg TTX**	(*n* = 5)	(*n* = 4)	(*n* = 3)	(*n* = 3)	(*n* = 3)
Body weight (g)	20.5 ± 0.2	18.6 ± 0.3	18.9 ± 0.5	19.1 ± 0.5	19.4 ± 0.4
Cumulative BW(g)	0.0	−1.9 ± 0.4	−1.6 ± 0.5	−1.3 ± 0.5	−1.1 ± 0.4

**Table 3:** Effect of chronic oral administration of TTX on feed consumption and feces and urine production during 24 h after the last TTX dose. Values are expressed as mean ± SEM, except for the dose of 75 µg/kg, where results represent the mean (*n* = 2).

Group/Analyzed Parameters (24 h in Metabolic Cages)	
**Control (*n* = 5)**	
Feed consumption (g)	5.2 ± 0.6
Feces (g)	1.5 ± 0.1
Urine (mL)	4.1 ± 0.8
**25 µg/kg TTX (*n* = 3)**	
Feed consumption (g)	3.3 ± 0.6
Feces (g)	1.3 ± 0.1
Urine (mL)	1.5 ± 1.1
**75 µg/kg TTX (*n* = 2)**	
Feed consumption (g)	4.6
Feces (g)	1.3
Urine (mL)	0.5
**125 µg/kg TTX (*n* = 3)**	
Feed consumption (g)	3.8 ± 0.6
Feces (g)	1.1 ± 0.3
Urine (mL)	0.5 ± 0.03 *

* *p* < 0.05 versus control values.

**Table 4:** Effect of repeated oral administration of TTX on urine color, clarity, specific gravity, protein, glucose, ketones, blood/hemoglobin, bilirubin, and urobilinogen from mice after 28 days of TTX dosage by oral gavage. Analytic parameters are expressed as mean ± SEM, except for the dose of 75 µg/kg, where results represent the mean (*n* = 2).

Group/Analyzed Parameters	Control (*n* = 5)	25 µg/kg TTX (*n* = 3)	75 µg/kg TTX (*n* = 2)	125 µg/kg TTX (*n* = 3)
Color	Pale yellow/ pale yellow/ amber/ pale yellow/ amber	Dark yellow/ pale yellow/ pale yellow	Dark yellow/ amber	Dark yellow/ amber/ dark yellow
Turbidity	Clear/ clear/ slightly cloudy/ slightly cloudy/ clear	Clear/ clear/ clear	Cloudy/ cloudy	Cloudy/ cloudy/ cloudy
Specific Gravity	>1050/1020/1032/ >1050/1020	>1050/ >1050/1026	>1050/ >1050	>1050/ >1050/ >1050
Urine protein (g/L)	0.3 ± 0.2	0.4 ± 0.3	3	1 ± 0.2
Glucose (mmol/L)	0	1.1 ± 0.9	3	7.7 ± 4.7
Ketones (mmol/L)	0	0	1.5	1 ± 0.5
Blood/haemoglobin (Ery/µL)	0	0	0	0
Bilirubin (µmol/L)	0	0	0	50
Urobilinogen (µmol/L)	0	0	0	52.3 ± 17.7

**Table 5:** TTX determination in feces, blood and organs of mice fed orally with 75 and 125 µg/kg of TTX. Determination of TTX in biological samples was performed in samples obtained 24 h after the last toxin dose. n.d.: not detected, LOD (blood) < 5.7 ng/mL, LOD (organs) < 3.4 ng/g, LOD (feces) < 3.3 ng/g.

TTX Dose (µg/kg)	Number of Animals	TTX Excreted by Feces (ng/g)	TTX in Blood	TTX in Organs
Control	3	n.d.	n.d.	n.d.
75	2	n.d.	n.d.	n.d.
125	3	145.9 ± 33.1	n.d.	n.d.

## Supplementary Materials

The following are available online at http://www.mdpi.com/2072-6651/11/2/96/s1, Figure S1: Effects of repeated dose exposure to TTX on blood ALT, AST, glucose, and cholesterol. Figure S2: Effects of repeated dose exposure to TTX on blood electrolyte levels and blood osmolality. Figure S3: Schematic diagram of the renal corpuscule indicating the main structures altered after chronic oral administration of TTX.
