# Supplementary material for: Chronic In Vivo Effects of Repeated Exposure to Low Oral Doses of Tetrodotoxin: Preliminary Evidence of Nephrotoxicity and Cardiotoxicity
Source: Toxins (Basel). 2019 Feb 6;11(2):96. doi: 10.3390/toxins11020096 (PMC6410189; doi:10.3390/toxins11020096)
Supplement: Supplementary file 1 [file toxins-11-00096-s001.pdf]

Supplementary Materials: Chronic In Vivo Effects of Repeated Exposure to Low Oral Doses of Tetrodotoxin: Preliminary Evidence of Nephrotoxicity and Cardiotoxicity

Andrea Boente-Juncal, Carmen Vale, Manuel Cifuentes, Paz Otero, Mercedes Camiña, Mercedes Rodriguez-Vieytes and Luis Miguel Botana

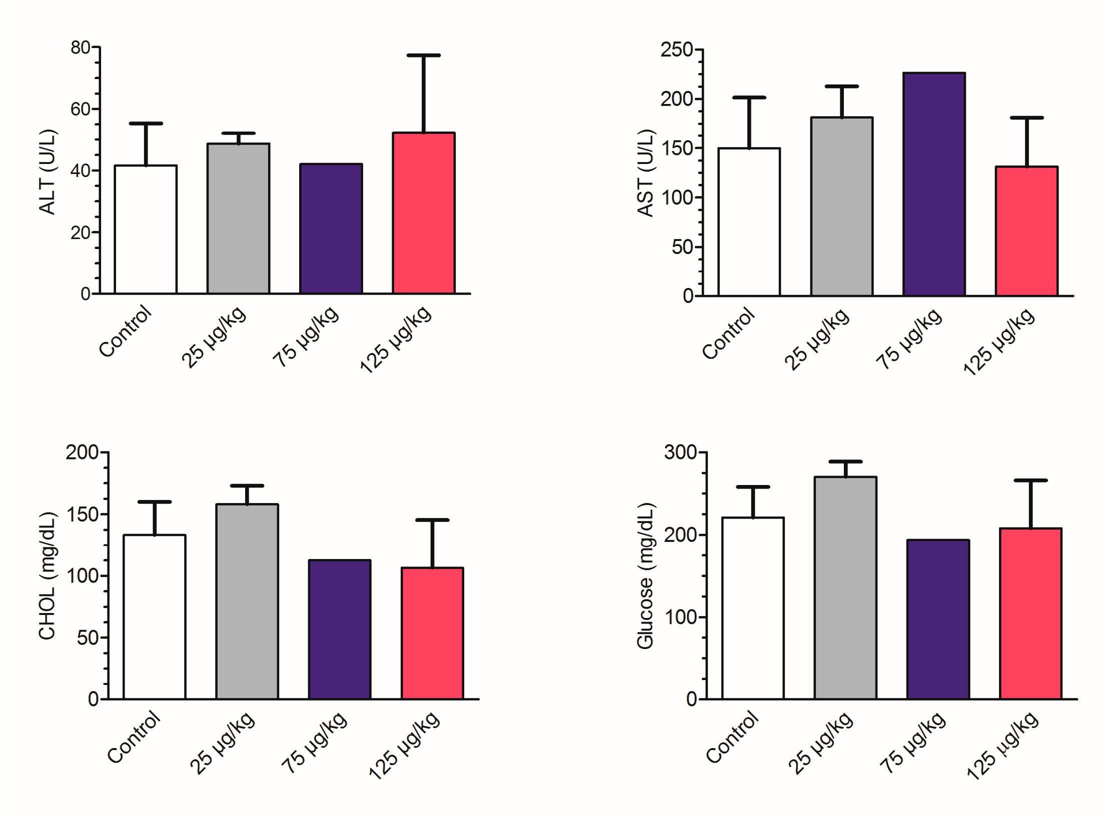

Figure S1. Effects of repeated dose exposure to TTX on blood ALT, AST, glucose, and cholesterol. Data are expressed as mean ± SEM of 3 to 5 determinations or mean only (n = 2) for the dose of 75 µg/kg.

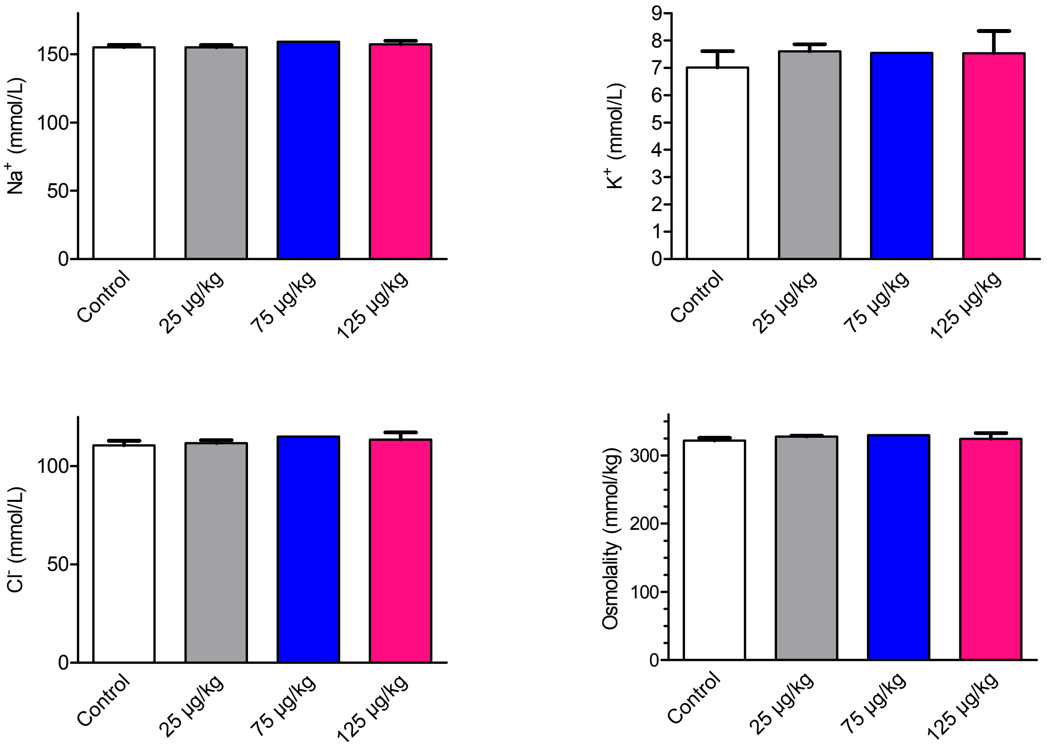

Figure S2. Effects of repeated daily oral exposure of mice to 125 µg/kg TTX on blood electrolyte levels and blood osmolality. Data are expressed as mean ± SEM of 3 to 5 determinations or mean only (n = 2) for the dose of 75 µg/kg.

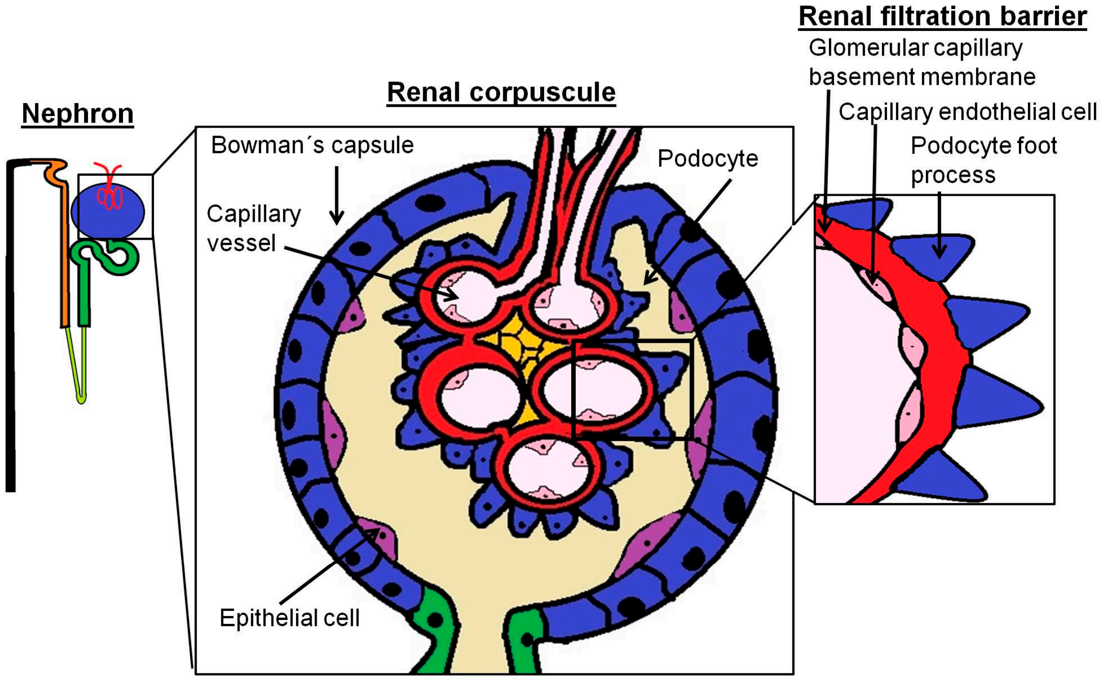

Figure S3. Schematic diagram of the renal corpuscule indicating the main structures altered after chronic oral administration of TTX.
